# Long-term impact of acute knee injury with hemarthrosis: Osteoarthritis incidence and patient-reported outcomes in a large consecutive cohort over 12 years

**DOI:** 10.1016/j.ocarto.2025.100700

**Published:** 2025-11-01

**Authors:** Anders Isacsson, Aleksandra Turkiewicz, Ola Olsson, Jamie S. Brown, Johan Hesslow, Patrik Bengtsson, Armaghan Mahmoudian, Richard B. Frobell, Jaanika Paemre, Martin Englund

**Affiliations:** a Clinical Epidemiology Unit, Orthopaedics, Department of Clinical Sciences Lund, Lund University, Lund, Sweden; b Department of Orthopaedics, Helsingborg Hospital, Helsingborg, Sweden; c Aleris Specialist Care, Orthopaedics, Ängelholm Hospital, Ängelholm, Sweden; d Movement Sciences and Health, University of West Florida, FL, USA; e Department of Clinical Sciences Lund, Orthopaedics, Lund University, Lund, Sweden; f Department of Radiology, Tartu University, Tartu University Hospital, Tartu, Estonia

**Keywords:** Osteoarthritis, Knee, Soft-tissue knee injury, Anterior cruciate ligament, Hemarthrosis, Outcome

## Abstract

**Objective:**

To estimate the 12-year risk of radiographic and/or symptomatic knee osteoarthritis following different MRI-confirmed soft-tissue knee injuries.

**Design:**

Prospective cohort study including 814 (70 ​%) of 1129 consecutive patients after acute knee trauma with hemarthrosis in a single hospital. Injuries were classified by sub-acute knee MRI. Follow-up comprised bilateral knee radiography and Knee injury and Osteoarthritis Outcome Score. The main outcomes were radiographic and symptomatic knee osteoarthritis. We estimated differences between injury types and injured versus contralateral knees.

**Results:**

The incidence proportion of radiographic knee osteoarthritis was 34 ​% (95 ​% confidence interval 30 to 37) in injured knees and 18 ​% (15, 20) in contralateral knees. Knees with hemarthrosis but no structural injury developed radiographic osteoarthritis and symptomatic osteoarthritis in 6 ​% and 4 ​%, respectively, as compared to 36 ​% and 21 ​% of those with structural injury, with risk ratios (95 ​% confidence interval) of 8 (3, 21) for radiographic osteoarthritis and 6 (2, 20) for symptomatic osteoarthritis. The risk ratio of tibiofemoral osteoarthritis in the injured knee compared to the contralateral knee was 2.2 (1.7, 2.8), with the highest ratio of 3.1 (2.1, 4.6) observed after combined anterior cruciate ligament (ACL) and meniscus injury. Overall, 81 ​% (78, 84) reported satisfaction with their knee function, though satisfaction was lower following patellar dislocation.

**Conclusions:**

Structural soft-tissue knee injury doubled the risk of radiographic and symptomatic knee osteoarthritis versus the contralateral knee over 12 years with significant variation based on injury type. ACL rupture combined with meniscus tear conferred the highest risk while hemarthrosis alone the lowest.

## 1 Introduction

It is well established that knee injuries increase the risk of developing knee osteoarthritis (OA) [[1], [2], [3], [4], [5], [6], [7], [8], [9], [10], [11]]. Acute knee injury with hemarthrosis, a strong indicator of soft-tissue injury, is a common reason to seek medical care [[12], [13], [14], [15]]. The annual incidence of traumatic knee hemarthrosis has been reported to 151 per 100,000 inhabitants [14]. In addition to acute bone marrow lesions (BML), the acutely injured knee often displays a combination of soft-tissue injuries. Anterior cruciate ligament (ACL) rupture, medial collateral ligament (MCL) injury, and meniscal tears are the most common such injuries in acute knee trauma with hemarthrosis, sometimes in isolation, but more often in combination [14,16,17]. In children, a lateral patellar dislocation (LPD) is the most common injury with a substantial risk of associated chondral injuries [[18], [19], [20]]. The lateral collateral ligament (LCL) and the posterior cruciate ligament (PCL) are less frequently injured. Occasionally, ligamentous structures and menisci remain intact, with only acute BMLs or no detectable signs of structural injury on MRI.

Although several studies have estimated the risk of post-traumatic knee OA, high-quality prospective evidence remains scarce, particularly regarding the long-term outcome of different structural injury combinations [1,21]. Systematic MRI-confirmation of different soft-tissue injuries is often lacking, sample sizes tend to be small, and suffer from substantial loss to follow-up. Consequently, our understanding of radiographic and self-reported outcomes in acutely injured knees, based on specific structural injury types, remains uncertain.

Thus, we conducted a 12-year follow-up of a large prospective cohort of consecutive patients with MRI-classified acute knee injuries, all presenting with hemarthrosis at a single hospital. The follow-up included standardized knee radiographs and validated self-reported outcome measures. Our objectives were to estimate the impact of different types of soft-tissue knee injuries on the development of both radiographic knee OA and symptomatic knee OA, as well as self-reported knee health and patient satisfaction. We also relate radiographic findings to the individuals’ contralateral knee.

## 2 Methods

### 2.1 Study cohort

As previously detailed, at baseline we included 1145 individual traumatic knee injuries in 1129 consecutive patients between 2002 and 2008 presenting with hemarthrosis at the emergency department or by referral to the acute out-patient knee clinic at Helsingborg Hospital, Sweden [14]. This hospital serves all paediatric as well as adult patient in a defined catchment area with a population of approximately 250,000 people. Hemarthrosis was either verified by aspiration (53 ​%) or an acute intra-articular effusion within 24 ​h after acute knee trauma [13]. Patients were excluded if there was no evidence of acute trauma, no evidence of rapid post-traumatic effusion, clear fluid on aspiration, delayed MRI until after the sub-acute period (defined as six weeks after injury), lacking medical records, or if they were already included in the study with the same knee. Knees with fractures other than osteochondral, avulsion or compression fractures were also excluded. All eligible patients had a sub-acute knee MRI within a median of 8 days (range, 0–42; interquartile range, 5–15 days) of the injury.

For long-term follow-up, the 1129 research subjects in the baseline cohort were contacted by telephone with at least three attempts and once per mail. Patients who could not be reached or did not attend twice for a scheduled appointment were considered lost to follow-up. For subjects that were included in the cohort for both their knees, the first entry was considered the index knee. Follow-up examination included radiographs of both knees, weight, height, and patient-reported outcome measures. Written informed consent was obtained, and the study was approved by the Swedish Ethical Review Authority (Dnr 2009/237 and 2015/250).

Some individuals from the cohort have also been included in the Knee Anterior Cruciate Ligament, Nonsurgical versus Surgical Treatment (KANON) trial, specifically those from the defined trial region (n ​= ​107/121, with follow-up n ​= ​87/107) and follow-up data has been reported [22]. Furthermore, follow-up data for the PCL injuries from the cohort (n ​= ​18) has also been presented earlier [23].

### 2.2 Classification of injuries based on knee MRI

Sub-acute MR images at baseline were originally read as part of the clinical routine according to the recommendations by Khanna et al., by radiologists experienced with knee MRI and transcribed into a predesigned protocol [14,24]. Observed injuries were classified into six main injury types (see also Suppl Fig 1).

a. Hemarthrosis alone: No structural injury except MCL or LCL grade 1 (edema only), i.e. only hemarthrosis as the intra-articular observation with or without BML.
b. LPD: Lateral patellar dislocation with or without concomitant cartilage injury potentially combined with other injuries but no ACL rupture.
c. Other injury: Structural injury in any combination of MCL or LCL grade 2–3, PCL any grade, cartilage injuries, osteochondral or compression fractures but no ACL rupture, LPD or meniscal tear.
d. Meniscus/not ACL: Meniscal tear but no ACL rupture (potentially combined with other injuries).
e. ACL/not meniscus: ACL rupture but no meniscal tear (potentially combined with other injuries).
f. ACL and meniscus: ACL rupture and meniscal tear (potentially combined with other injuries).

Low-frequency structural injuries in knees without concomitant ACL injury, meniscal tears or LPD were considered as one group (i.e. Other injury) (Suppl Fig 1).

### 2.3 Follow-up knee radiography

Standardized plain weight-bearing bilateral radiographs were obtained in three views: posteroanterior frontal plane (Schuss view) and sagittal plane in semi-flexion of the tibiofemoral (TF) compartment and axial plane of the patellofemoral (PF) compartment [25]. Radiographs were graded according to the Osteoarthritis Research Society International atlas by a specialist in musculoskeletal radiography (JP) who was blinded to clinical data [26,27]. The presence of radiographic OA was assessed by evaluating joint space narrowing (JSN) and osteophytes in each compartment, i.e. medial TF, lateral TF, medial PF and lateral PF, respectively, using a categorical scale: 0 ​= ​normal, 1 ​= ​mild change, 2 ​= ​moderate change and 3 ​= ​severe change.

### 2.4 Patient-reported Outcome Measures

Knee-related health was assessed using the Knee injury and Osteoarthritis Outcome Score (KOOS), activity level with Tegner Activity Scale and knee satisfaction through a study-specific questionnaire incorporating validated measurements previously used in musculoskeletal disorder research (Suppl Table 1) [[28], [29], [30], [31], [32], [33], [34]]. The questionnaire included questions on general illness since the injury, hospitalizations and additional knee injuries.

### 2.5 Definitions and reliability of radiographic and symptomatic knee osteoarthritis

Radiographic knee OA was defined as the presence of at least one osteophyte grade ≥2, JSN grade ≥2, or a sum score of JSN and osteophyte grades ≥2 within at least one compartment (medial TF, lateral TF and/or PF) [35]. Tibiofemoral OA was present if the definition of OA was fulfilled in either the medial or lateral compartment. Intra-observer variability was calculated from repeated readings of 40 individuals (80 knees) with a Cohen's Kappa (bootstrap 95 ​% confidence intervals) of 0.89 (0.76, 1.00) for TF OA and 0.81 (0.63, 0.98) for PF OA. The definition approximates Kellgren and Lawrence grade 2 or higher for the TF joint (Cohen's kappa 0.90 in our material) [36]. Total knee arthroplasty (n ​= ​2) was considered as both TF and PF OA. An individual was considered to have symptomatic knee OA if the criteria for radiographic OA in the injured knee was fulfilled *and* had a self-reported symptomatic knee based on KOOS scores, as previously defined (Suppl Fig 2) [35].

### 2.6 Statistical analysis

Descriptive statistics are presented as medians with interquartile range. Cumulative incidences of radiographic knee OA and symptomatic knee OA, respectively, were calculated as incidence proportions with 95 ​% logit transformed confidence intervals (CIs). We assumed that knee OA was not present in either knee at time of injury since the median age was 26 (IQR, 17–35) and only 8 ​% (88/1129) were older than 44 years at the time of injury. None was older than 64 years.

Analysis of the risk of radiographic OA in the injured compared to the contralateral knee in the same individual, accounting for both measured and unmeasured person-level confounders (e.g. age, sex, body mass, physical activity level etc.), was made. The two knees were analyzed as a matched pair by a fixed effects Poisson regression model, thus including only individuals with at least one outcome (OA). Risk ratios (RR) were calculated with 95 ​% CIs with the contralateral knee as reference category. Additionally, we adjusted for earlier (collected from charts at baseline) or new (reported at follow-up) injuries.

Comparisons between individuals with different injury types and hemarthrosis alone in their injured knee were conducted using a Poisson regression model with robust standard errors to estimate RR and 95 ​% CIs. Due to the low incidence of OA in the hemarthrosis alone group, an adjusted regression model comparing other injury groups to this group was not feasible. Thus, crude estimates with adjustments for logarithm of time to follow-up are presented. However, comparison between injury groups and the miscellaneous group of other injury, i.e. other than ACL, meniscus or LPD, was made with additional adjustments for age, sex, sports-related injury, and body mass index. Also, earlier knee injury to the index or contralateral knee as noted from the medical records at baseline and new knee injury to either knee after the index injury as reported by the patient at the time of follow-up were adjusted for.

Since KOOS, TAS and knee satisfaction score exhibit ceiling effects, a Tobit model (linear regression for censored data) was applied, adjusting for the confounders mentioned above, and results reported as mean differences with 95 ​% CIs.

The analyses were conducted including all persons with non-missing outcome data, under missing at random assumption [37]. We provide CIs as inferential measures, due to the observational nature of the study with no a priori sample size calculation. The original study was designed to include all eligible patients presenting between the years 2002 and 2008, when standardized MRI and clinical routines were used, and no sample size calculation with the current outcomes in mind were made. Calculations were made using Stata 18 (StataCorp. 2023. *Stata Statistical Software: Release 18*. College Station, TX: StataCorp LLC).

## 3 Results

### 3.1 Descriptive characteristics

**Fig. 1:**
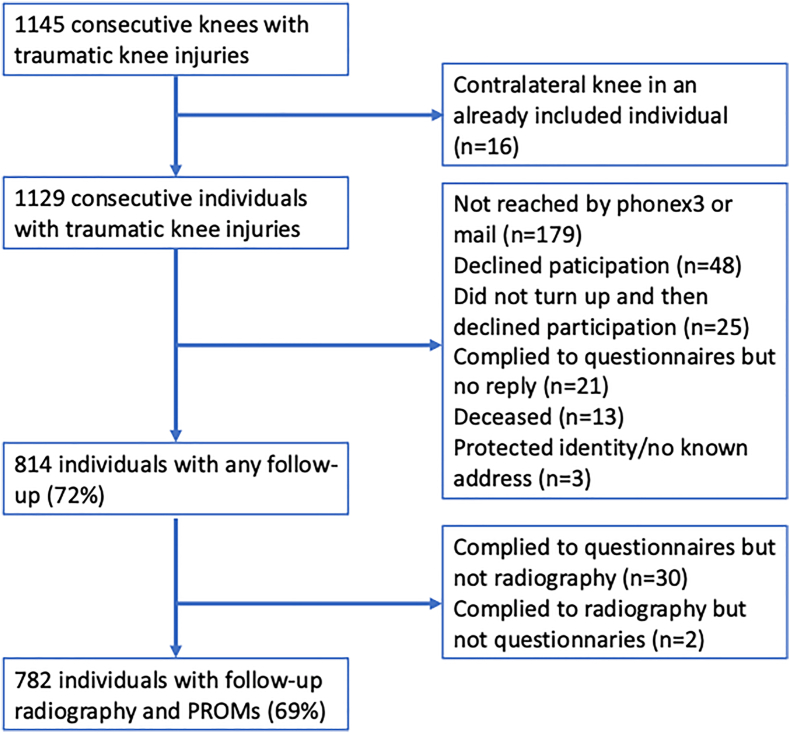
Flowchart of the study cohort and loss to follow-up by reason. PROMs, patient-reported outcome measures.

Seventy-two percent of the patients (n ​= ​814) from the baseline cohort adhered to follow-up, and 69 ​% (n ​= ​782) had a full follow-up at a median of 12 years (IQR 11–14, range 8–18) including bilateral knee radiography, weight, height, and patient-reported outcome measures (Fig. 1).

Median age at follow-up was 39 years (IQR 31–50; range 19–78). No major differences were observed in sex distribution, age at injury, injury during sports, or noted earlier injury in either knee between the baseline and the follow-up cohorts. New injuries were most frequently reported after LPD and least frequently after meniscal tear/not ACL. Injury to the ACL occurred most frequently during sport, whilst LPD least frequently (Table 1 and Suppl Table 2).

**Table 1:** Descriptive data of the baseline cohort, the follow-up cohort and the subgroups by injury type.

	Baseline cohort	Follow-up cohort	Follow-up cohort, injury types					
			Hemarthrosis alone	LPD	Other injury	Meniscus not ACL	ACL not meniscus	ACL and meniscus
Individuals, n (% of group baseline)	1129	782 (69 ​%)	70 (56 ​%)	124 (66 ​%)	67 (70 ​%)	70 59 ​%)	197 (74 ​%)	254 (76 ​%)
Median age at injury, years (IQR; range)	26 (17–35; 6–64)	26 (18–37; 6–64)	17 (14–24; 6–58)	18 (15–24; 10–47)	31 (21–40; 12–63)	34 (22–43; 13–63)	30 (22–39; 14–62)	29 (21–38; 12–64)
Female sex	36 ​%	39 ​%	37 ​%	44 ​%	37 ​%	19 ​%	47 ​%	36 ​%
Right knee	52 ​%	51 ​%	41 ​%	56 ​%	43 ​%	50 ​%	49 ​%	55 ​%
Injury during sports	72 ​%	72 ​%	63 ​%	52 ​%	61 ​%	60 ​%	85 ​%	82 ​%
Earlier injury index knee	24 ​%	24 ​%	19 ​%	27 ​%	24 ​%	26 ​%	18 ​%	29 ​%
Earlier injury contralat.	14 ​%	16 ​%	7 ​%	16 ​%	12 ​%	19 ​%	18 ​%	16 ​%
New injury index knee,		30 ​%	23 ​%	54 ​%	28 ​%	15 ​%	24 ​%	32 ​%
New injury contralat.		23 ​%	24 ​%	34 ​%	19 ​%	37 ​%	14 ​%	22 ​%
Median time to follow-up, years (IQR; range)		12.1 (10.9–14,0; 7.9–17.5)	13.9 (12.1–15.5; 10.5–17.5)	10.2 (8.8–11.9; 7.9–17.2)	12.3 (11.2–14.6; 8.9–16.8)	14.1 (11.8–15.5; 10.5–17.2)	12.0 (11.0–13.5; 8.8–17.4)	12.3 (11.0–14.1; 8.9–17.2)
Median BMI at follow-up (IQR, range)		26 (24–29; 16–47)	25 (22–28; 20–40)	26 (23–31; 18–47)	27 (25–30; 18–44)	27 (24–29; 18–40)	26 (24–29; 18–41)	26 (24–29; 16–43)

Injury types: Hemarthrosis alone, no structural injury. LPD, LPD but no ACL rupture. Other injury, other structural injuries including medial or lateral collateral ligament grade 2–3, posterior cruciate ligament any grade, cartilage injuries or fractures but no ACL rupture, meniscal tear or LPD. Meniscus/not ACL, meniscal tear but no ACL rupture. ACL/not meniscus, ACL rupture but no meniscal tear. ACL and meniscus, ACL rupture in combination with meniscus tear.

Earlier injury refers to injury before index injury at baseline according to retrospective data from charts collected per protocol. New injury refers to new injury collected as self-reported data at follow-up visit. IQR, interquartile range. Contralat., contralateral knee. LPD, lateral patellar dislocation. ACL, anterior cruciate ligament rupture. BMI, body mass index. IQR, interquartile range. For more information on baseline cohort see Suppl Table 1.

### 3.2 Radiographic knee OA and symptomatic knee OA

The incidence proportion of radiographic knee OA was 34 ​% (95 ​% CI 30, 37) in the injured and 18 ​% (95 ​% CI 15, 20) in the contralateral knee (Table 2). At follow-up, 20 ​% reported a symptomatic knee along with radiographic signs of OA in the affected knee. No essential sex-dependent differences were observed.

**Table 2:** Incidence proportions of radiographic knee osteoarthritis (OA) and symptomatic knee OA, respectively^a^.

Knee OA definition Full cohort	Injured knee, n	Incidence proportion Index knee, % (95 ​% CI)	Contralateral. knee, n	Incidence proportion Contralateral. knee, % (95 ​% CI)
Radiographic knee OA	263/784	34 (30, 37)	138/783	18 (15, 20)
Radiographic TF OA	195/784	25 (22, 28)	89/783	11 (9, 14)
Radiographic PF OA	199/784	25 (22, 29)	113/783	14 (12, 17)
Symptomatic knee OA	155/780	20 (17, 23)	N/a	N/a
Males				
Radiographic knee OA	170/483	35 (31, 40)	87/483	18 (15, 22)
Radiographic TF OA	122/483	25 (22, 29)	53/483	11 (8, 14)
Radiographic PF OA	131/483	27 (23, 31)	74/483	15 (12, 19)
Symptomatic knee OA	99/481	21 (17, 24)	N/a	N/a
Females				
Radiographic knee OA	93/301	31 (26, 36)	51/300	17 (13, 22)
Radiographic TF OA	73/301	24 (20, 29)	36/300	12 (9, 16)
Radiographic PF OA	68/301	23 (18, 28)	39/300	13 (10, 17)
Symptomatic knee OA	56/301	19 (15, 23)	N/a	N/a

TF, tibiofemoral. PF, patellofemoral. N/a, not applicable. KOOS, Knee injury and Osteoarthritis Outcome Score.

a There were two individuals with missing KOOS data and one who opted to have radiography of the injured knee only.

**Fig. 2:**
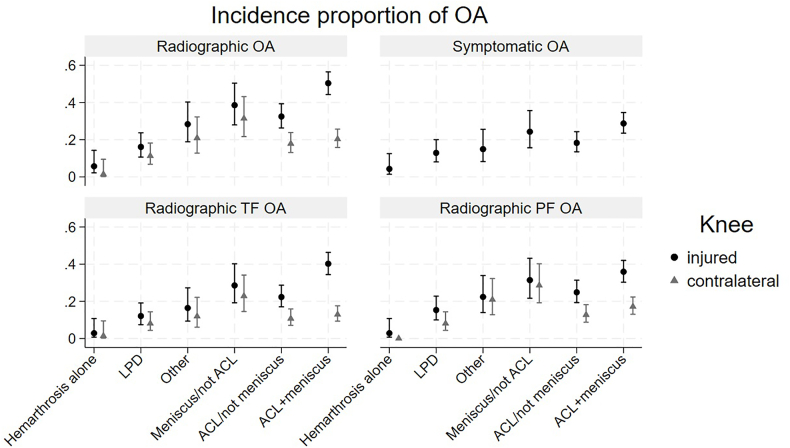
Incidence proportions of radiographic knee osteoarthritis (OA) and symptomatic knee OA by injury group with 95 ​% CIs and comparisons between index and contralateral knees. Radiographic OA, OA in any compartment. Symptomatic OA, radiographic OA in combination with KOOS scores equivalent with a knee considered symptomatic. KOOS, Knee injury and Osteoarthritis Outcome Score. PF, patellofemoral. TF, tibiofemoral. Please see Table 1 for description of injury types and other abbreviations, Table 3 for count values per subgroup, and suppl Fig. 2 for definition of a symptomatic knee.

The combination of ACL rupture and meniscal tear had the worst outcome regarding development of radiographic OA compared to the contralateral knee with an incidence proportion of 50 ​% (95 ​% CI 44, 56) in the injured knee and 20 ​% (95 ​% CI 16, 26) in the contralateral knee. Similar results were observed for ACL rupture without meniscal tear (32 ​% (95 ​% CI 26, 39) vs 18 ​% [95 ​% CI 13, 24]) while lower risk differences between the two knees were seen in other groups (Fig. 2 and Table 3).

**Table 3:** Incidence proportions of radiographic and symptomatic osteoarthritis.

Injury type, n (proportion, 95 ​% CI)	Sympt OA index	OA index	TF OA index	PF OA index	OA contra	TF OA contra	PF OA contra
Hemarthrosis alone - No structural injury	3/70 (4 ​%, 1-12)	4/70 (6 ​%, 2-14)	2/70 (3 ​%, 0-10)	2/70 (3 ​%, 0-10)	1/70 (1 ​%, 0-9)	1/70 (1 ​%, 0-9)	0/70 (0 ​%, -)
LPD	16/124 (13 ​%, 8-20)	20/124 (16 ​%, 11-24)	15/124 (12 ​%, 7-19)	19/124 (15 ​%, 10-23)	14/124 (11 ​%, 7-18)	10/124 (8 ​%, 4-14)	10/124 (8 ​%, *4-14*)
Other injury	10/67 (15 ​%, 8-26)	19/67 (28 ​%, *19-40*)	11/67 (16 ​%, 9-27)	15/67 (22 ​%, *14-34*)	14/67 (21 ​%, *13-32*)	8/67 (12 ​%, 6-22)	14/67 (21 ​%, *13-32*)
Meniscus/not ACL	17/69 (24 ​%, *16-36*)	27/70 (39 ​%, *28-50*)	20/70 (29 ​%, *19-40*)	22/70 (31 ​%, *22-43*)	22/70 (31 ​%, *22–43*	16/70 (23 ​%, *14-34*)	20/70 (29 ​%, *19-40*)
ACL/not meniscus	35/196 (18 ​%, *13-24*)	64/197 (32 ​%, ***26-39***)	44/197 (22 ​%, ***17-29***)	49/197 (25 ​%, ***19-31***)	35/196 (18 ​%, *13-24*)	21/196 (11 ​%, 7-16)	25/196 (13 ​%, *9-18*)
ACL and meniscus	74/254 (29 ​%, *24-35*)	129/256 (50 ​%, ***44-56***)	103/256 (40 ​%, ***34-46***)	92/256 (36 ​%, ***30-42***)	52/256 (20 ​%, *16-26*)	33/256 (13 ​%, 9-18)	44/256 (17 ​%, *13-22*)
All injuries except hemarthrosis alone	152/710 (21 ​%, *18–25*)	259/714 (36 ​%, ***33–40***)	193/714 (27 ​%, ***24–30***)	197/714 (28 ​%, ***24–31***)	137/713 (19 ​%, 16-22)	88/713 (12 ​%, 10-15)	113/713 (16 ​%, 13-19)
Meniscus and/or ACL	126/519 (24 ​%, *21-28*)	220/523 (42 ​%, ***38-46***)	167/523 (32 ​%, ***28-36***)	163/523 (31 ​%, ***27-35***)	109/522 (21 ​%, 17-25)	70/522 (13 ​%, 11-17)	89/522 17 ​%, 14-21)
All injuries	155/780 (20 ​%, 17-23)	263/784 (34 ​%, **30**–**37**)	195/784 (25 ​%, **22**–**28**)	199/784 (25 ​%, **22**–**29**)	138/783 (18 ​%, 15-20)	89/783 (11 ​%, 9-14)	113/783 (14 ​%, 12-17)

Due to missing data, OA for ACL/not meniscus in the contralateral knee was calculated on 196 individuals. Symptomatic OA for Meniscus/not ACL was calculated on 69 individuals, for ACL/not meniscus on 196 individuals, and for ACL and meniscus on 254 individuals.

Bold indicates where the 95 ​% CI does not overlap with the contralateral knee and italics where the 95 ​% CI does not overlap with the group of knees with hemarthrosis alone and no structural injury.

OA, Osteoarthritis in any compartment. Symptomatic OA, osteoarthritis in combination with KOOS scores equivalent with symptoms. KOOS, knee injury and osteoarthritis outcome score. PF, patellofemoral. TF, tibiofemoral. Contra, contralateral knee. CI, confidence interval. See Table 1 for description of injury types and other abbreviations.

Regarding symptomatic knee OA, all injury types had notably worse outcome than hemarthrosis alone, with highest incidence proportion in ACL rupture and meniscal tear (29 ​% [95 ​% CI 24, 35]) (Fig. 2 and Table 3).

All structural injuries resulted in higher incidence proportions of radiographic knee OA (36 ​%, 95 ​% CI 33, 40) compared to hemarthrosis alone (6 ​%, 95 ​% CI 2, 14). The PF and TF compartments seemed to be equally afflicted regardless of injury type. These results also applied to the contralateral knee. Surprisingly, LPD cases had less PF OA, 15 ​% (95 ​% CI 10, 23), than all other injury types (Fig. 2 and Table 3).

Two of 70 individuals (3 ​%, 95 ​% CI 0, 10) with hemarthrosis alone showed signs of TF OA as compared to 103 of 256 (40 ​%, 95 ​% CI 34, 46) after combined ACL rupture and meniscal tear (Table 3).

The RR of radiographic knee OA after soft-tissue knee injury with hemarthrosis in the within-person matched pair analysis was 1.9 (95 ​% CI 1.5, 2.3) compared to the contralateral knee. However, the RR was dependent on type of injury (Table 4). In a sensitivity analysis we adjusted for earlier injury to either knee before inclusion or new injury during the time to follow-up without any essential changes to the estimates (Suppl Table 3).

**Table 4:** Risk ratios of radiographic and symptomatic knee OA in *within-person* comparisons of injured versus contralateral knee in individuals with different injury types and *between-persons* comparisons of individuals with specific injury types to individuals with hemarthrosis alone.

	Within person		Between persons	
	versus contralateral knee		versus hemarthrosis alone	
	Radiographic knee OA			
	RR	95 ​% CI	RR crude	95 ​% CI
Any structural injury^a^	1.9	1.5, 2.3	7.8	2.9, 20.9
LPD	1.4	0.7, 2.8	4.1	1.4, 12.2
Other injury	1.4	0.7, 2.7	5.7	2.0, 16.1
Meniscus/not ACL	1.2	0.7, 2.2	6.8	2.5, 18.7
ACL/not meniscus	1.8	1.2, 2.7	6.7	2.5, 18.2
ACL and meniscus	2.5	1.8, 3.4	10.1	3.8, 27.0
	**Radiographic TF OA**			
Any structural injury^a^	2.2	1.7, 2.8	11.7	2.9, 47.3
LPD	1.5	0.7, 3.3	6.3	1.4, 28.2
Other injury	1.4	0.6, 3.4	6.6	1.5, 29.2
Meniscus/not ACL	1.3	0.7, 2.4	10.1	2.4, 42.0
ACL/not meniscus	2.1	1.3, 3.5	9.3	2.3, 38.2
ACL and meniscus	3.1	2.1, 4.6	16.2	4.0, 65.5
	**Radiographic PF OA**			
Any structural injury^a^	1.7	1.4, 2.2	12.2	3.0, 49.0
LPD	1.9	0.9, 4.1	8.6	2.0, 37.6
Other injury	1.1	0.5, 2.2	9.2	2.2, 39.5
Meniscus/not ACL	1.1	0.6, 2.0	11.2	2.7, 46.1
ACL/not meniscus	1.9	1.2, 3.1	10.7	2.6, 43.8
ACL and meniscus	2.1	1.5, 3.0	14.9	3.7, 60.1
	**Radiographic and symptomatic knee OA**			
Any structural injury^a^	n/a		6.4	2.0, 20.2
LPD	n/a		5.2	1.5, 18.6
Other injury	n/a		4.2	1.2, 15.0
Meniscus/not ACL	n/a		5.8	1.7, 19.0
ACL/not meniscus	n/a		5.4	1.7, 17.6
ACL and meniscus	n/a		8.1	2.6, 25.8

TF, tibiofemoral, PF, patellofemoral. OA, osteoarthritis. RR, risk ratio. CI, confidence interval. Please see Table 1 for description of injury types and other abbreviations.

a Hemarthrosis alone not counted as a structural soft-tissue injury.

In the between-persons analysis, comparing individuals with different injuries to those with hemarthrosis alone, the crude RR for radiographic OA was 7.8 (95 ​% CI 2.9, 20.9). While elevated RRs for radiographic and symptomatic knee OA were seen across all injury groups, the large CIs reflect the low frequency of OA in knees with hemarthrosis alone (Table 4).

We further estimated adjusted RRs comparing knees with any of the injuries LPD, ACL rupture and/or meniscal tears to other injuries. However, only knees that suffered a combined ACL rupture and meniscal tear displayed a clearly higher frequency of both radiographic OA and symptomatic OA with adjusted RRs of 1.9 (95 ​% CI 1.3, 2.7) and 2.2 (95 ​% CI 1.3, 3.6), respectively (Suppl Table 4).

### 3.3 Patient-reported outcome and satisfaction

**Fig. 3:**
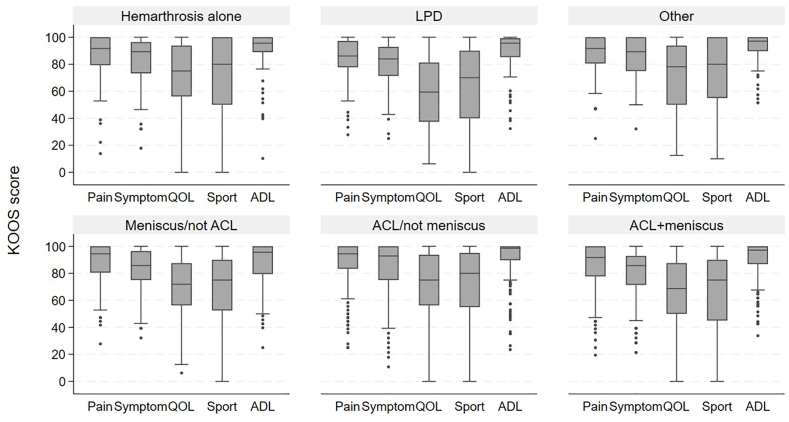
KOOS subscales by injury type. Box plot with medians and interquartile range shown as boxes. KOOS, Knee injury and Osteoarthritis Outcome Score. ADL, activities of daily living. QOL, quality of life. Please see Table 1 for description of injury types and other abbreviations.

LPD was associated with less satisfaction with the knee at follow-up (Fig. 3) with lower scores in the KOOS subscales for knee-related function in sports and recreational activities (−13.3 [95 ​% CI -24.4, −2.2]) as well as lower knee-related quality of life (QoL) by 15 points (−14.8 [95 ​% CI -23.8, −5.8]) as compared to hemarthrosis alone (Suppl Tables 5a and 5b). Likewise, the physical activity level measured in Tegner Activity Scale was lower after LPD (−1.0 [95 ​% CI -1.6, −0.39]) versus hemarthrosis alone.

Patients with a combined ACL rupture and meniscal tear also scored worse than hemarthrosis alone for the KOOS subscale of QoL with a mean difference of 10 points (95 ​% CI -18.1, - 2.3), whilst no essential differences were observed for other injury groups. All observed KOOS scores had CIs that excluded differences larger than 10 points and virtually no differences remained after additional adjustment for earlier or new injuries and body mass index (Suppl Tables 5a and 5b).

Four out of five subjects were satisfied with their knee function (Suppl Table 6).

## 4 Discussion

Further knowledge regarding outcomes after acute knee injury has been suggested as one of the most important research priorities for soft tissue knee injuries [38]. In our large prospective cohort of consecutive patients with acute knee injuries and hemarthrosis, the development of knee OA was strongly associated with the type of initial injury as identified on MRI. Among all injury types, ACL rupture combined with meniscal tear was the most detrimental. The risk of radiographic tibiofemoral OA in the affected knee was three times higher in the paired analysis compared to the contralateral knee after a median of 12 years with this common injury combination. Compared to patients with hemarthrosis alone, the risk of radiographic OA in those with an ACL rupture and meniscal tear was 10 times higher while the risk of symptomatic knee OA was eight times higher. These findings of large differences in risk between the paired analyses and the between-persons analyses indicate that, in addition to the injury itself, person-specific risk factors have a significant role in the development of knee OA.

Twelve years after soft-tissue knee injury, one in three individuals developed radiographic OA in the injured knee while one in five had the combination of radiographic and symptomatic OA. Additionally, one in five developed radiographic OA in the contralateral knee. Osteoarthritis is a prevalent cause of disability, with the knee being the most affected site, impacting one-third of the global population over the age of 70 [39]. Our study confirms earlier studies that knee injury is a major contributor to OA development, which has been estimated to a 4–6 fold increased risk in both the general population and young adults [1,21,40]. Moreover, both ACL ruptures and meniscal tears are recognised as strong risk factors for OA development and progression [1]. The trajectory of OA progression after different types of knee injuries appears to be variable in this material. Individuals with ACL injury combined with meniscal tear are likely having the fastest rate of progression to OA, even though rates of progression should be interpreted with caution given that no repeated measurements have been undertaken.

Of the 70 individuals with hemarthrosis alone, i.e. those who did not exhibit any structural injury except BML at index MRI, only 3 ​% developed TF OA in the index knee and 1 ​% in the contralateral knee. Individuals with a combined ACL injury and meniscal tear on index MRI, on the other hand, developed tibiofemoral OA in 40 ​% of injured knees and 13 ​% of contralateral knees. Even though hemarthrosis was not quantified, the bleeding itself did not seem to generate any substantial incidence of OA which has been speculated [41,42]. Acute hemarthrosis has also been hypothesized to have a protective effect through enhanced meniscus healing [43]. Either genetic or behavioral factors, i.e. being a person that exposes the knees to risk of injury, appear to play a larger role than hemarthrosis in this respect.

In our within-person between-knee comparison that adjusts for person-level confounding, ACL rupture with meniscal tear tripled the risk of developing radiographic OA after 12 years whilst an ACL rupture without meniscal tear doubled it. Interestingly, a torn meniscus *without* concomitant ACL rupture did not confer any strongly increased risk as compared to the contralateral knee within the 12-year time frame. However, the between-persons comparison versus hemarthrosis alone yielded a highly increased risk. Genetical or behavioral risk factors may carry a larger risk of developing OA, attenuating the specific effect of a knee injury. This points towards large inter-individual differences in risk that, to our knowledge, have not been previously reported. Interestingly though, high genetic contribution to both meniscal tears and ACL ruptures have been described [44,45].

Patellofemoral OA is often considered to be caused by increased chondral stress due to anatomical risk factors and instability where LPD has been reported to be a significant risk factor [46,47]. Surprisingly, in our study LPD was associated with less patellofemoral OA than all other structural soft-tissue knee injuries except hemarthrosis alone. However, these patients were younger at injury than others and may develop radiographic OA at a later stage. Also, this was the youngest injury group and older age at knee injury has been reported to confer a greater risk of post-traumatic OA [21,48].

KOOS values from a representative Danish population cohort showed higher median scores for all subscales than this cohort [49]. Most notable difference was in the QoL subscale with a median score of 88 in the age group 18–29 years compared to 59 (IQR, 44) after LPD in this study. Overall, four out of five were satisfied with their knee function at 12 years after injury, which has been associated with return to pre-injury sports level, high knee-related self-efficacy and QoL [34].

The main strength of our study was the prospective inclusion of consecutive soft-tissue knee injuries presenting at a single hospital in a well-defined catchment area with the aim to include all traumatic knee injuries with hemarthrosis in the population. Furthermore, all diagnoses were ascertained by MRI and there is a relatively low loss to follow-up for a study of this type (approximately 30 ​%). Still, there are noteworthy limitations that we would like to highlight. We did not systematically assess radiographic knee OA status before index injury but expected that the prevalence would be low due to the typical young age at inclusion. Of the 782 individuals with full follow-up, 57 ​% had plain knee radiography at the time of index injury. In the radiologists’ clinical reports, osteophytes were noted in 4 ​% and JSN in 0.4 ​%. Another limitation is that we considered all meniscal tears as one entity, regardless of tear type, if the lateral and/or medial meniscus was torn, or the extent of the tears. For instance, certain root tears may result in a more significant loss of meniscus function than minor stable meniscal tears seen on MRI [50]. Further, data on earlier injuries to either knee as collected from the medical records at baseline and self-reported new injuries between baseline and follow-up was available and adjusted for, but no information on type or severity. Residual confounding in the models adjusted for earlier or new injuries cannot be excluded.

Patients were treated according to standard of care during the study period which included both non-surgical and surgical management of, e.g., ACL injuries. Unfortunately, we currently have limited information on what grounds the individuals received a certain treatment and also the specific treatments received during the follow-up period.

There was a tendency for older individuals and individuals with ACL rupture to adhere to follow-up to a greater extent. We cannot exclude the possibility that those with more symptoms at the time of follow-up were more inclined to participate. Also, the follow-up procedure was staged between different injury sub-groups. This led to slightly different follow-up times on average, but with large overlap between the groups, and we aimed to account for it in the regression models. Especially, the LPD group had follow-up earlier than the others.

In conclusion, this study provides high-quality prospective evidence on how *different* MRI-confirmed soft-tissue knee injuries contribute to the risk of developing knee OA and impact knee-related health 12 years after acute knee injury. Our findings also suggest that knee injury alone may not increase the risk of OA as much as previously thought once person-level factors are accounted for. While the injury pattern remains important, the data indicates that individual susceptibility plays a profound role.

## Author contributions

AI, OO, RF, AT and ME contributed to the study design and analysis plan. AI, JB, JH and PB recruited and examined the patients. JP interpreted and graded the plain radiographs at follow-up. AT was responsible for the statistical analyses. AM contributed with critical revision of the article for intellectual content. AI drafted the first version of the manuscript. All authors commented on scientific content and approved the final manuscript.

## Role of the funding source

This study has been funded by the Stig and Ragna Gorthon Foundation, Helsingborg, Sweden, The 10.13039/501100004359Swedish Research Council, Österlund Foundation, Gustaf V 80-Year Birthday Foundation, 10.13039/501100006075Greta and Johan Kock Foundation, Governmental Funding of Clinical Research within National Health Service (ALF), the 10.13039/501100007949Swedish Rheumatism Association, and the Foundation for People with Movement Disability in Skåne. None of the funding sources had any involvement in this study, from conception to manuscript.

## Conflict of interest

ME reports consultancy for Grünenthal Sweden AB, Key2 Compliance AB, and Genascence. AT is associate editor for statistics at the Osteoarthritis and Cartilage journal. All other authors report no conflicts of interests.
