# Supplementary material for: Long-term impact of acute knee injury with hemarthrosis: Osteoarthritis incidence and patient-reported outcomes in a large consecutive cohort over 12 years
Source: Osteoarthr Cartil Open. 2025 Nov 1;7(4):100700. doi: 10.1016/j.ocarto.2025.100700 (PMC12685512; doi:10.1016/j.ocarto.2025.100700)
Supplement: Multimedia component 1 [file mmc1.pdf]

Long-term impact of acute knee injury with hemarthrosis: Osteoarthritis incidence and patient-reported outcomes in a large consecutive cohort over 12 years

Supplementary material

**Hemarthrosis alone (n=70):**	1 MCL* + LCL* + BML, 4 MCL* + BML, 2 isolated MCL*, 1 LCL* + BML, 36 isolated BML, and 26 with no findings
**LPD (n=124):**	7 MCL, 1 LCL, 4 MM, 7 LM and, 1 MM + LM, but excludes 3 with typical signs of LPD on MRI with concomitant ACL rupture as this was considered the main injury
**Other injury (n=67):**	4 isolated PCL, 1 PCL + MCL + comp, 1 PCL + MCL, 20 isolated MCL, 5 MCL + cart, 3 MCL + comp, 1 LCL + cart + OCF, 2 cart + OCF, 4 cart + comp, 12 isolated cart, 14 isolated comp
**Meniscus/not ACL (n=70):**	37 MM, 25 LM, 8 MM + LM. In combination with 4 PCL, 3 PCL + MCL, 9 MCL, 1 LCL
**ACL/not meniscus (n=197):**	3 PCL, 4 PCL + MCL, 1 PCL + MCL + LCL, 52 MCL, 3 MCL + LCL, 4 LCL, 2 LPD
**ACL and meniscus (n=254):**	114 MM, 69 LM, 71 MM + LM. These occurred in combination with 4 PCL (1 PCL + MCL + cart + comp, 1 PCL + MCL, 1 PCL + LCL, 1 isolated PCL), 62 MCL (1 MCL + LCL, 1 MCL + LCL + cart, 1 MCL + LCL + comp, 4 MCL + cart + imp, 6 MCL + cart, 25 MCL + comp, 24 isolated MCL), 7 LCL (1 LCL + comp, 1 LCL + cart, 5 isolated LCL), and 1 LPD

Supplemental Figure 1. Details of included injuries in each subgroup of injury type after classification.
MRI, magnetic resonance imaging. BML, bone marrow lesion. MCL, medial collateral ligament injury. LCL, lateral collateral ligament injury. LPD, lateral patellar dislocation. MM, medial meniscal tear. LM, lateral meniscal tear. ACL, anterior cruciate ligament rupture. PCL, posterior cruciate ligament rupture. OCF, osteochondral fracture. Cart, cartilage injury. Comp, compression fracture.
*MCL/LCL Grade 1, all other collateral ligament injuries (MCL/LCL) were grade 2 or 3.

Supplemental Table 1. Knee satisfaction score and additional questions*		
1. Are you satisfied with your present knee function?		
Alternatives:	Yes/No	
2. If not, do you consider your treatment as a failure?		
Alternatives:	Yes/No	
3. Are you satisfied with your current knee function?”		
Alternatives:	10-point Likert scale from 1 “Not satisfied at all” to 10 “Very satisfied”	
4. If you were to spend the rest of your life with your knee function just the way it has been over the last week, would you feel …?		
	<u>*Satisfied*</u>	<u>*Dissatisfied*</u>
Alternatives:	“Happy”, “Satisfied”, “Mostly satisfied”,	“Mixed feelings”, “Mostly dissatisfied”, “Dissatisfied” and “Unhappy”.
Additional questions on general or knee-related illness since the index injury		
Have you been seriously ill since your knee injury?		
Alternatives:	Yes/No/I don’t remember	
If yes, where and when did you receive care for your illness?		
Do you, or have you been, on any medication since your knee injury?		
Alternatives:	Yes/No – If yes, please note which ones	
Which knee did you injure when included in the study?		
Alternatives:	Right/Left	
Have you injured *the same* knee since the index injury?		
Alternatives:	Yes/No – If yes, please note which injury	
Have you injured *the other* knee since the index injury?		
Alternatives:	Yes/No – If yes, please note which injury	
If yes, where and when did you receive care for your injury/injuries?		

*Questions and alternatives have been abbreviated

Definition of symptomatic osteoarthritis:

A knee was classified as symptomatic if

1. the KOOS subscale for knee-related Quality of Life (QoL) and
2. at least two of the remaining four subscales
3. had scores equal to or less than the threshold obtained when at least half of the questions in a subscale were answered with one or more steps decrease from the best response on the 5-point Likert scale.

This calculates into scores indicating a symptomatic knee after conversion to a 0-100 scale (worst to best outcome), in the subscales for knee-related

- symptoms ≤85.7,
- pain ≤86.1,
- ADL ≤86.8,
- sports/recreation ≤85.0 and,
- QoL ≤87.5.

Supplemental Figure 2. Definition of symptomatic osteoarthritis according to Englund et al, 2003.

Supplemental Table 2. Descriptive data on baseline and follow-up cohorts with subgroups by injury type

	Baseline cohort							Follow-up cohort										
	Individuals, n	Age at index injury (median, IQR; range)	Sex female (%)	Injured knee right (%)	Injury during sports	Earlier injury index knee, ratio (ratio md)	Earlier injury contra knee, ratio (ratio md)	Number of patients (f-u ratio)	Age at index injury (median, IQR; range)	Sex female (%)	Injured knee right (%)	Injury during sports	Time to follow-up, years (median, IQR; range)	Earlier injury index knee (ratio md)	Earlier injury contra knee (ratio md)	New injury index knee (ratio md)	New injury contra knee (ratio md)	BMI at follow-up (median; IQR, range; md)
Entire cohort	1129	26 (17-35; 6.2-64.4)	36%	52%	72%	24%	14%	782 (69%)	26 (18-37; 6.3-64.4)	39%	51%	72%	12.1 (10.9-14,0; 7.9-17.5)	24%	16%	30%	23%	26 (23.8-29.2; 16.3-46.8; n=3)
Injury type																		
Hemarthrosis alone	124	15.8 (13.8-23.4; 6.2-57.6)	37%	46%	68%	16% (16%)	9% (16%)	70 (56%)	17.4 (14.0-23.9; 6.3-57.6)	37%	41%	63%	13.9 (12.1-15.5; 10.5-17.5)	19% (17%)	7% (19%)	23% (0%)	23% (0%)	25 (22-28; 20-40)
LPD	187	17.2 (17.6-23.5; 9.2-47.0)	40%	52%	53%	31% (8%)	14% (12%)	124 (66%)	18.0 (14.7-24.1; 10.6-47.0)	44%	56%	52%	10.2 (8.8-11.9; 7.9-17.2)	27% (7%)	16% (11%)	52% (2%)	34% (2%)	26 (23-31; 18-47; n=1)
Other structural injury	96	28.8 (20.1-40.5; 12.3-63.3)	32%	49%	66%	21% (23%)	9% (28%)	67 (70%)	31.3 (20.5-40.8; 12.3-63.3)	37%	43%	61%	12.3 (11.2-14.6; 8.9-16.8)	24% (19%)	12% (25%)	28% (0%)	19% (0%)	27 (25-30; 18-44)
Meniscus/not ACL	119	27.0 (17.7-41.6; 9.6-63.3)	19%	49%	66%	22% (22%)	16% (28%)	70 (59%)	34.2 (22.1-43.5; 13.0-63.3)	19%	50%	60%	14.1 (11.8-15.5; 10.5-17.2)	26% (29%)	19% (34%)	14% (4%)	36% (3%)	27 (24-29; 18-40; n=1)
ACL/not meniscus	268	29.1 (20.6-38.9; 13.9-62.7))	44%	50%	84%	18% (19%)	17% (24%)	197 (74%)	30.3 (21.6-39.2; 14.0-62.7)	47%	49%	85%	12.0 (11.0-13.5; 8.8-17.4)	18% (19%)	18% (23%)	24% (1%)	14% (1%)	26 (24-29; 18-41)
ACL and meniscus	335	28.2 (20.7-37.7; 10.2-64.4)	34%	56%	79%	30% (15%)	15% (22%)	254 (76%)	28.6 (20.9-38.1; 12.0-64.4)	36%	55%	82%	12.3 (11.0-14.1; 8.9-17.2)	29% (15%)	16% (23%)	31% (2%)	21% (2%)	26 (24-29; 16-43; n=1))

See Suppl Fig 1 for description of injury types and other abbreviations.
Earlier injury refers to knee injury before index injury at baseline according to retrospective data from charts collected per protocol. New injury refers to new knee injury collected as self-reported data at follow-up visit. IQR, interquartile range. BMI, body mass index. F-u, follow-up. Contra knee, contralateral knee. Md, missing data.

Supplemental Table 3. Risk ratios in a matched pair analysis of radiographic osteoarthritis with 95 % confidence intervals in the injured knee compared to the contralateral knee with adjustment for known previous injury before index trauma.

	**Any radiographic knee OA, crude**		**After adjustment for earlier injury***		**After adjustment for earlier or new injury***	
	RR	95% CI	RR	95% CI	RR	95% CI
Injured knee	1.90	1.54, 2.33	1.85	1.45, 2.34	1.88	1.53, 2.32
Any structural injury	1.88	1.53, 2.32	1.83	1.44, 2.33	1.87	1.51, 2.31
Hemarthrosis alone#						
LPD	1.43	0.72, 2.83	1.37	0.66, 2.85	1.42	0.71, 2.82
Other injury	1.36	0.68, 2.71	1.35	0.62, 2.92	1.14	0.56, 2.32
Meniscus/not ACL	1.23	0.70, 2.15	1.14	0.58, 2.26	1.32	0.74, 2.34
ACL/not meniscus	1.80	1.19, 2.72	1.93	1.19, 3.14	1.89	1.24, 2.87
ACL and meniscus	2.48	1.80, 3.42	2.33	1.61, 3.38	2.42	1.74, 3.37
	**Radiographic TF knee OA**					
Injured knee	2.19	1.71, 2.82	2.35	1.73, 3.20	2.23	1.72, 2.90
Any structural injury	2.19	1.70, 2.82	2.40	1.75, 3.29	2.24	1.72, 2.91
Hemarthrosis alone#						
LPD	1.50	0.67, 3.34	1.83	0.76, 4.38	1.53	0.67, 3.45
Other injury	1.37	0.55, 3.42	1.44	0.51, 4.07	1.01	0.39, 2.64
Meniscus/not ACL	1.25	0.65, 2.41	1.23	0.55, 2.78	1.47	0.74, 2.91
ACL/not meniscus	2.10	1.25, 3.52	2.54	1.32, 4.88	2.21	1.29, 3.78
ACL and meniscus	3.12	2.11, 4.62	3.40	2.11, 5.49	3.21	2.13, 4.86
	**Radiographic PF knee OA**					
Injured knee	1.75	1.39, 2.21	1.64	1.2, 2.14	1.74	1.38, 2.21
Any structural injury	1.73	1.38, 2.19	1.61	1.23, 2.11	1.73	1.36, 2.19
Hemarthrosis alone#						
LPD	1.90	0.88, 4.09	1.71	0.77, 3.81	1.89	0.87, 4.08
Other injury	1.07	0.52, 2.22	1.02	0.45, 2.34	0.89	0.42, 1.89
Meniscus/not ACL	1.10	0.60, 2.02	0.95	0.45, 1.99	1.18	0.64, 2.19
ACL/not meniscus	1.92	1.18, 3.11	1.87	1.07, 3.29	2.07	1.26, 3.39
ACL and meniscus	2.09	1.46, 2.99	1.93	1.28, 2.93	2.03	1.40, 2.94

TF, tibiofemoral, PF, patellofemoral. OA, osteoarthritis, RR, risk ratio. CI, confidence interval. Injured knee, all knee injuries including hemarthrosis alone (no structural injury). Any structural injury, all injuries except hemarthrosis alone. See Fig 1 and Table 1 for description of injury types and other abbreviations.

*Here only persons with non-missing data on earlier or new injury are included.

#Hemarthrosis alone not possible to estimate due to low counts, almost no OA in this group (n=4).

Supplemental Table 4. Between-persons analyses comparing the specific injury types to other structural injury than LPD, ACL injury and/or meniscal tear. Estimates are risk ratios of OA in the index knee from a Poisson regression model with robust standard errors adjusted for time to follow-up, age, sex and if injury during sports participation with additional adjustment for earlier or new injury in the index knee and BMI.

Injury type	RR (95%CI), adjusted for time to follow-up, age, sex and injury at sports		Also adjusted for earlier or new injury to index knee		Also adjusted for BMI	
	**Any radiographic knee OA**					
	RR	95% CI	RR	95% CI	RR	95% CI
Other injury	Reference category					
Any structural injury vs other injury	1.44	1.00, 2.09	1.43	1.00, 2.03	1.50	1.06, 2.10
LPD	1.08	0.63, 1.86	0.99	0.57, 1.69	0.99	0.58, 1.67
Meniscus/not ACL	1.07	0.68, 1.68	1.10	0.71, 1.70	1.19	0.77, 1.82
ACL/not meniscus	1.23	0.82, 1.84	1.24	0.84, 1.83	1.30	0.89, 1.90
ACL and meniscus	1.86	1.28, 2.70	1.82	1.27, 2.61	1.90	1.34, 2.69
	**Radiographic TF knee OA**					
Other injury	Reference category					
Any structural injury vs other injury	1.88	1.08, 3.27	1.85	1.09, 3.15	1.97	1.20, 3.25
LPD	1.26	0.60, 2.65	1.11	0.53, 2.31	1.11	0.55, 2.26
Meniscus/not ACL	1.42	0.75, 2.69	1.48	0.81, 2.73	1.63	0.91, 2.92
ACL/not meniscus	1.46	0.80, 2.66	1.48	0.83, 2.62	1.60	0.93, 2.75
ACL and meniscus	2.57	1.47, 4.49	2.50	1.46, 4.27	2.67	1.61, 4.42
	**Radiographic PF knee OA**					
Other injury	Reference category					
Any structural injury vs other injury	1.41	0.93, 2.15	1.40	0.93, 2.10	1.50	1.03, 2.19
LPD	1.53	0.85, 2.75	1.41	0.79, 2.52	1.41	0.80, 2.47
Meniscus/not ACL	1.03	0.63, 1.70	1.05	0.65, 1.71	1.18	0.74, 1.88
ACL/not meniscus	1.22	0.77, 1.94	1.23	0.78, 1.93	1.32	0.87, 2.02
ACL and meniscus	1.70	1.10, 2.61	1.67	1.10, 2.55	1.78	1.20, 2.64
	**Radiographic and symptomatic knee OA**					
Other injury	Reference category					
Any structural injury vs other injury	1.66	0.93, 2.94	1.62	0.94, 2.77	1.77	1.07, 2.94
LPD	1.70	0.81, 3.57	1.45	0.70, 3.00	1.45	0.71, 2.99
Meniscus/not ACL	1.21	0.61, 2.39	1.28	0.68, 2.41	1.43	0.78, 2.62
ACL/not meniscus	1.37	0.73, 2.58	1.40	0.77, 2.52	1.54	0.88, 2.69
ACL and meniscus	2.04	1.14, 3.68	1.96	1.13, 3.41	2.15	1.28, 3.63

TF, tibiofemoral, PF, patellofemoral. OA, osteoarthritis, RR, risk ratio. CI, confidence interval. BMI, bone mass index. Any structural injury, all knee injuries except the reference category. See Fig 1 and Table 1 for description of injury types and other abbreviations.

Supplemental Table 5a. Between-persons analysis of KOOS comparing the injury types. Mean differences from a tobit regression model adjusted for age, sex, if injury during sports participation and logarithm of follow-up time and thereafter additionally adjusted for earlier or new injury to index knee and BMI at follow-up.

PROM	Non missing (n)	Median [IQR]	Adjusted mean difference (points)	95% CI	Additionally adjusted mean difference (points)	95% CI
**KOOS symptom - all**	812	86 [21]				
Hemarthrosis alone (ref)	72	89 [23]	0.00		0.00	
LPD	132	84 [21]	-3.75	-10.52, 3.03	1.17	-5.62, 7.96
Other injury	70	89 [25]	1.65	-5.74, 9.05	4.15	-3.11, 11.40
Meniscus/not ACL	73	86 [21]	-2.50	-9.77, 4.77	-1.75	-8.93, 5.44
ACL/not meniscus	199	93 [25]	0.06	-6.19, 6.30	1.04	-5.09, 7.16
ACL and meniscus	266	86 [21]	-4.74	-10.67, 1.20	-2.50	-8.35, 3.35
**KOOS pain - all**	806	92 [22]				
Hemarthrosis alone (ref)	72	92 [21]	0.00		0.00	
LPD	131	86 [19]	-6.03	-13.15, 1.09	-0.75	-7.82, 6.31
Other injury	70	92 [19]	2.29	-5.53, 10.10	5.24	-2.38, 12.87
Meniscus/not ACL	73	94 [19]	1.02	-6.68, 8.72	2.57	-5.01, 10.14
ACL/not meniscus	197	94 [17]	1.80	-4.81, 8.41	3.06	-3.36, 9.47
ACL and meniscus	263	92 [22]	-1.63	-7.88, 4.62	0.89	-5.22, 7.00
**KOOS adl - all**	811	97 [12]				
Hemarthrosis alone (ref)	72	96 [11]	0.00		0.00	
LPD	132	96 [14]	-3.78	-10.65, 3.09	1.20	-5.58, 7.97
Other injury	70	97 [10]	3.41	-4.08, 10.89	5.82	-1.43, 13.06
Meniscus/not ACL	73	96 [21]	-0.17	-7.56, 7.21	1.42	-5.82, 8.65
ACL/not meniscus	199	99 [10]	2.74	-3.63, 9.11	4.01	-2.15, 10.18
ACL and meniscus	265	97 [13]	1.20	-4.84, 7.24	3.60	-2.27, 9.46
**KOOS sport - all**	809	75 [50]				
Hemarthrosis alone (ref)	72	80 [50]	0.00		0.00	
LPD	132	70 [50]	-13.30	-24.42, -2.18	-4.42	-15.35, 6.51
Other injury	70	80 [45]	4.52	-7.60, 16.64	9.26	-2.42, 20.95
Meniscus/not ACL	72	75 [38]	-3.18	-15.13, 8.77	-0.83	-12.43, 10.77
ACL/not meniscus	198	80 [40]	-0.72	-10.99, 9.55	1.49	-8.37, 11.35
ACL and meniscus	265	75 [45]	-7.98	-17.73, 1.76	-3.15	-12.58, 6.29
**KOOS qol - all**	809	75 [38]				
Hemarthrosis alone (ref)	72	75 [38]	0.00		0.00	
LPD	132	59 [44]	-14.76	-23.76, -5.76	-8.15	-17.05, 0.75
Other injury	70	78 [44]	-0.43	-10.20, 9.34	2.45	-6.99, 11.90
Meniscus/not ACL	72	72 [31]	-5.57	-15.25, 4.11	-5.05	-14.49, 4.39
ACL/not meniscus	198	75 [38]	-3.88	-12.17, 4.41	-2.88	-10.88, 5.11
ACL and meniscus	265	69 [38]	-10.18	-18.06, -2.29	-7.37	-15.04, 0.30

BMI, bone mass index. PROM, patient-reported outcome measure. KOOS, knee osteoarthritis and outcome score. ADL, activities of daily living. QoL, Quality of Life. See Fig 1 and Table 1 for description of injury types and other abbreviations.

Supplemental Table 5b. Between-persons analysis of Tegner and Knee satisfaction score comparing the injury types. Mean differences from a tobit regression model adjusted for age, sex, if injury during sports participation and logarithm of follow-up time and thereafter additionally adjusted for earlier and new injury to index knee and BMI at follow-up.

**PROM**	Non missing (n)	Median [IQR]	Adjusted Mean difference (points)	95% CI	Additionally adjusted Mean difference (points)	95% CI
**Tegner**						
Hemarthrosis alone (ref)	72	4 [4]	0.00		0.00	
LPD	131	3 [2]	-1.02	-1.65, - 0.39	-1.14	-1.79, -0.49
Other injury	70	3 [2]	-0.58	-1.26, 0.10	-0.56	-1.25, 0.12
Meniscus/not ACL	73	3 [3]	-0.44	-1.12, 0.23	-0.57	-1.25, 0.12
ACL/not meniscus	200	4 [4]	-0.27	-0.84, 0.31	-0.29	-0.87, 0.29
ACL and meniscus	264	4 [4]	-0.50	-1.05, 0.05	-0.53	-1.09, 0.02
**Knee satisfaction score**						
Hemarthrosis alone (ref)	72	8 [3]	0.00		0.00	
LPD	132	7 [3]	-0.90	-1.75, -0.05	-0.22	-1.06, 0.63
Other injury	69	8 [3]	-0.09	-1.01, 0.84	0.28	-0.62, 1.18
Meniscus/not ACL	73	8 [3]	-0.21	-1.13, 0.71	-0.04	-0.94, 0.86
ACL/not meniscus	200	8 [2]	-0.08	-0.86, 0.70	0.10	-0.67, 0.86
ACL+meniscus	261	8 [3]	-0.51	-1.26, 0.23	-0.17	-0.90, 0.56

BMI, bone mass index. PROM, patient-reported outcome measure. Tegner, Tegner activity scale. Knee satisfaction score, “Are you satisfied with your current knee function?” on a 10-point Likert scale from 1 “Not satisfied at all” to 10 “Very satisfied”. CI, confidence interval. See Fig 1 and Table 1for description of injury types and other abbreviations.

Supplemental Table 6. Satisfaction with knee function at a median of 12 years after different knee injuries as answered by the question “If you were to spend the rest of your life with your knee function just the way it has been over the last week, would you feel …?”

	Satisfied (n)	Dissatisfied (n)	Satisfied (%)	95% CI
Hemarthrosis alone (n=70)	56	14	80	69-89
LPD (n=124)	89	35	72	63-79
Other injury (n=67)	61	6	91	82-97
Meniscus/not ACL (n=70)	57	13	81	70-90
ACL/not meniscus (n=197)	163	34	83	77-88
ACL and meniscus (n=254)	206	48	81	76-86
All (n=782)	632	150	81	78-84

“Satisfied” includes answering: “Happy”, “Satisfied” or “Mostly satisfied”. Dissatisfied includes answering: “Mixed feelings”, “Mostly dissatisfied”, “Dissatisfied” or “Unhappy”. CI, Confidence interval. See Table 1 for description of injury types and other abbreviations.
